# Modulation of Na^+^/K^+^ ATPase Activity by Hydrogen Peroxide Generated through Heme in *L*. *amazonensis*

**DOI:** 10.1371/journal.pone.0129604

**Published:** 2015-06-12

**Authors:** Nathália Rocco-Machado, Daniela Cosentino-Gomes, José Roberto Meyer-Fernandes

**Affiliations:** 1 Institute of Medical Biochemistry Leopoldo de Meis, Federal University of Rio de Janeiro (UFRJ), CCS, Cidade Universitária, Ilha do Fundão, 21941-590, Rio de Janeiro, RJ, Brazil; 2 Institute of National Science and Technology of Structural Biology and Bioimage (INCTBEB), CCS, Cidade Universitária, Ilha do Fundão, 21941-590, Rio de Janeiro, RJ, Brazil; Instituto Nacional de Salud Pública, MEXICO

## Abstract

*Leishmania amazonensis *is a protozoan parasite that occurs in many areas of Brazil and causes skin lesions. Using this parasite, our group showed the activation of Na^+^/K^+^ ATPase through a signaling cascade that involves the presence of heme and protein kinase C (PKC) activity. Heme is an important biomolecule that has pro-oxidant activity and signaling capacity. Reactive oxygen species (ROS) can act as second messengers, which are required in various signaling cascades. Our goal in this work is to investigate the role of hydrogen peroxide (H_2_O_2_) generated in the presence of heme in the Na^+^/K^+^ ATPase activity of *L*. *amazonensis*. Our results show that increasing concentrations of heme stimulates the production of H_2_O_2_ in a dose-dependent manner until a concentration of 2.5 μM heme. To confirm that the effect of heme on the Na^+^/K^+^ ATPase is through the generation of H_2_O_2_, we measured enzyme activity using increasing concentrations of H_2_O_2_ and, as expected, the activity increased in a dose-dependent manner until a concentration of 0.1 μM H_2_O_2_. To investigate the role of PKC in this signaling pathway, we observed the production of H_2_O_2_ in the presence of its activator phorbol 12-myristate 13-acetate (PMA) and its inhibitor calphostin C. Both showed no effect on the generation of H_2_O_2_. Furthermore, we found that PKC activity is increased in the presence of H_2_O_2_, and that in the presence of calphostin C, H_2_O_2_ is unable to activate the Na^+^/K^+^ ATPase. 100 μM of Mito-TEMPO was capable of abolishing the stimulatory effect of heme on Na^+^/K^+^ ATPase activity, indicating that mitochondria might be the source of the hydrogen peroxide production induced by heme. The modulation of *L*. *amazonensis* Na^+^/K^+^ ATPase by H_2_O_2_ opens new possibilities for understanding the signaling pathways of this parasite.

## Introduction

*Leishmania* spp. are a group of protozoa from the Trypanosomatidae family that causes a variety of diseases that can manifest in a cutaneous or visceral form depending on the species of *Leishmania* [1,2]. *Leishmania amazonensis* is the major etiological agent of cutaneous or diffuse cutaneous leishmaniasis in South America [3,4]. The protozoan presents two distinct morphological and functional forms, nonflagellate intracellular amastigotes living inside the macrophages of the vertebrate host and motile flagellate promastigotes that live in the alimentary tract of the blood-sucking insect vector [5]. As a bloodstream form, promastigotes of *Leishmania* are constantly exposed to free heme, a pro-oxidant molecule with signaling capacity that comes from the digestion of hemoglobin in the midgut of the insect vector [6–9]. Despite being considered deleterious to the cell in excess, reactive oxygen species (ROS) play an important role in cell signaling [10–14]. Hydrogen peroxide (H_2_O_2_) is generated by the dismutation of the superoxide anion radical (O_2_˙ˉ) and can cross cell membranes more easily. At low concentrations it may play an important role in cell signaling pathways through oxidation of specific target molecules [10,11,15].

Heme is a metalloporphyrin that performs many functions as a prosthetic group of different hemoproteins involved in oxidative metabolism, oxygen storage and transport, and signal transduction [16]. *Leishmania*, as well as other trypanosomatid protozoa, lack several enzymes in the heme biosynthetic pathway and thus depend on extracellular heme uptake for survival [17,18]. It has been shown that there is a high binding affinity for heme in the membrane of *L*. *amazonensis* promastigotes [19], *L*. *infantum* amastigotes [20] and a specific uptake of the heme analogue magnesium protoporphyrin IX (MgPPIX) in *L*. *donovani* [21]. In 2012 a gene in *L*. *amazonensis* was identified that has homology to HRG-4, a *Caenorhabditis elegans* gene encoding a heme transporter in the plasma membrane [22]. This gene was given the name *Leishmania* heme response-1 (LHR1) [23]. The heme uptake by LHR1 was shown to be involved in *L*. *amazonensis* virulence [24].

Our group showed in 2010 that heme stimulates Na^+^/K^+^ ATPase activity through a signaling pathway involving protein kinase C (PKC) in *L*. *amazonensis* [25]. Na^+^/K^+^ ATPase is a pump that catalyzes the ATP-dependent exchange of 3 Na^+^ for 2 K^+^ across the cell membrane, creating an electrochemical gradient, and is present in *Leishmania* species [26–28]. The PKC family consists of serine/threonine kinases that are involved in a variety of signals. Studies show evidence of the existence of specific PKC-like activity in *Leishmania* [25,27,29,30]. Knowing that heme is a pro-oxidant molecule and the importance of H_2_O_2_ in signal transduction, our goal in this work is to investigate if heme can promote an increase in the H_2_O_2_ production by *L*. *amazonensis* and if this H_2_O_2_ is involved in the activation of Na^+^/K^+^ ATPase.

## Materials and Methods

### 1. Reagents

All reagents were purchased from E. Merck (São Paulo, Brazil) or Sigma—Aldrich (St. Louis, MO). Deionized distilled water was obtained from a Milli-Q system of resins (Millipore Corp., Bedford, MA) and was used in the preparation of all solutions.

### 2. Microorganisms

The MHOM/BR/75/Josefa strain of *L*. *amazonensis* [31] was used throughout this study. The MHOM/BR/75/Josefa strain was kindly supplied by Dr. Marcos André Vannier-Santos from Fundação Oswaldo Cruz, Centro de Pesquisa Gonçalo Muniz, Salvador, Bahia, Brazil. Promastigotes have been maintained in our laboratory in axenic culture using Warren’s medium [32] supplemented with 10% heat-inactivated fetal bovine serum at 22°C. Parasites were harvested at the stationary phase, sixth day of growth by centrifugation, washed twice and maintained at room temperature in a buffer consisting of 116 mM NaCl, 5.4 mM KCl, 5.5 mM D-glucose, and 50 mM Hepes—tri(hydroxymethyl)aminomethane (Hepes—Tris), pH 7.2.

### 3. Cell proliferation curve

1 x 10^6^ cells were added in Warren medium with 10% fetal bovine serum. Every 24 hours, aliquots of 50 μl were taken from each flask culture and the cell density was estimated daily by counting aliquots in a Neubauer chamber hemocytometer. The number of cells of each day was obtained by the weighted average of triplicate in three different curves with different cell suspensions.

### 4. Cell lysate preparations

The cells were washed twice in 50 mM Hepes—Tris buffer, pH 7.2, in the absence of Na^+^ and K^+^ and counted in a Neubauer chamber. Cell lysates from MHOM/BR/75/Josefa strain of *L*. *amazonensis* [31] were prepared by three freeze—thaw cycles in liquid nitrogen until sufficient cells were obtained to yield 5 mg/mL protein (5 x 10^8^ cells/mL). The total protein concentration was determined by the method of Lowry *et al* (1951) using bovine serum albumin as a standard [33].

### 5. Na^+^/ K^+^ ATPase activity assay

Na^+^/ K^+^ ATPase activity was measured in a reaction medium containing 20 mM Hepes—Tris pH 7.2, 10 mM MgCl_2_, 5 mM ATP, [γ^32^P]ATP (specific activity of approximately 10^4^ Bq/nmol ATP), 120 mM NaCl and 30 mM KCl in a final volume of 0.1 mL. ATPase activity was assayed by measuring the hydrolysis of [γ^32^]ATP as described previously [27]. The reaction was initiated by the addition of cell lysate (0.5 mg protein/mL) and stopped after 1 h by addition of 1.0 mL of ice-cold 25% charcoal in 1.0 M HCl. The tubes were then centrifuged at 1500 *g* for 10 min at 4°C. Aliquots (0.5 mL) of the supernatants containing the released ^32^Pi (inorganic phosphate) were transferred to scintillation vials containing 9.0 mL of scintillation fluid (2.0 g PPO in 1 L of toluene). The [^32^P]Pi released was measured using a scintillation counter. Spontaneous hydrolysis of [γ^32^P]ATP was measured simultaneously by adding protein to some tubes after the addition of activated charcoal. The Na^+^/ K^+^ ATPase activity was calculated as the difference between [^32^Pi] released in the absence and in the presence of 1 mM ouabain [34].

### 6. Hydrogen peroxide production assay

The production of H_2_O_2_ by living cells of *L*. *amazonensis* promastigotes was determined fluorimetrically by the method of Amplex red oxidation (Invitrogen) in the presence of horseradish peroxidase (Invitrogen). With Amplex Red it is possible to evaluate the release of H_2_O_2_ by the cell under real physiological conditions [35]. A reaction medium (final volume 0.2 mL) containing 100 mM sucrose, 20 mM KCl, 50 mM Tris-HCl, pH 7.2, and 1.7 mM of Amplex Red was used. Cells (5 x10^8^/mL) were incubated for 20 minutes under different conditions, as indicated in the figure legends, and the reaction was triggered with 13.4 μL of 100 U/mL horseradish peroxidase. Fluorescence was monitored at excitation and emission wavelengths of 563 ± 5 nm and 587 ± 5 nm, respectively. After 20 min of reaction, H_2_O_2_ production was determined using a standard curve with known quantities of H_2_O_2_. The results were normalized by cell number and expressed in pmol H_2_O_2_ x 10^–8^ cells.

### 7. Cell viability assay

*L*. *amazonensis* viability in the presence of 50 μM heme and 2.5 μM H_2_O_2_ was evaluated by the quantitative colorimetric MTT [3-(4,5-dimethylthiazol-2-yl)-2,5-diphenyl tetrazolium bromide)] assay. The mitochondrial electron chain converts MTT to formazan, and a decrease in the concentration of MTT indicates toxicity to the cell [36]. Cells (5 x10^8^/mL) were incubated in a 96-well plate with a reaction medium (final volume 0.2 mL) containing 100 mM sucrose, 20 mM KCl, 50 mM Tris-HCl, pH 7.2, and 50 μM heme or 2.5 μM H_2_O_2_ for 1 hour, and then the MTT labeling reagent (final concentration, 0.20 mg/mL) was added to each well. After a 2-h incubation, DMSO was added to dissolve the formazan crystals and obtain a homogeneous blue solution suitable for measurement of the absorbance at 590 nm. Parasite viability (%) was calculated regarding the control. We used 1% Triton X-100 as a positive control.

### 8. Protein kinase C activity assay

*L*. *amazonensis* intact promastigotes (5x10^8^/mL) were incubated, in different conditions, in a reaction medium containing 100 mM sucrose, 20 mM KCl and 50 mM Tris-HCl, pH 7.2, for 20 minutes. After this time, cells were washed twice in Hepes 50 mM Mes-buffer and lysed in liquid nitrogen for 15 minutes. PKC activity was assayed in the presence of 4 mM Hepes-Tris, pH 7.0, 0.4 mM MgCl_2_, 1 mM CaCl_2_, 0.36 mg/μL Neurogranin (specific substrate for PKC), 25 nM ATP and 40 μg of lysed cells in a final volume of 40 μl. The reaction was triggered by adding 40 μl of the Kinase-Glo luminescent kit [37], and after 10 minutes at 30°C cultures were placed in a luminometer (Promega Multi Glomax Junior).

### 9. Statistical analysis

All experiments were performed in triplicate, and similar results were obtained from at least three different cell suspensions. Data were analyzed statistically by Student’s t-test or a one-way ANOVA followed by the Tukey test using Prism computer software (GraphPad Software Inc., San Diego, CA, USA). A result was considered to be statistically significant when *p* < 0.05.

## Results

### 1. H_2_O_2_ generation during *Leishmania amazonensis* proliferation

**Fig 1:**
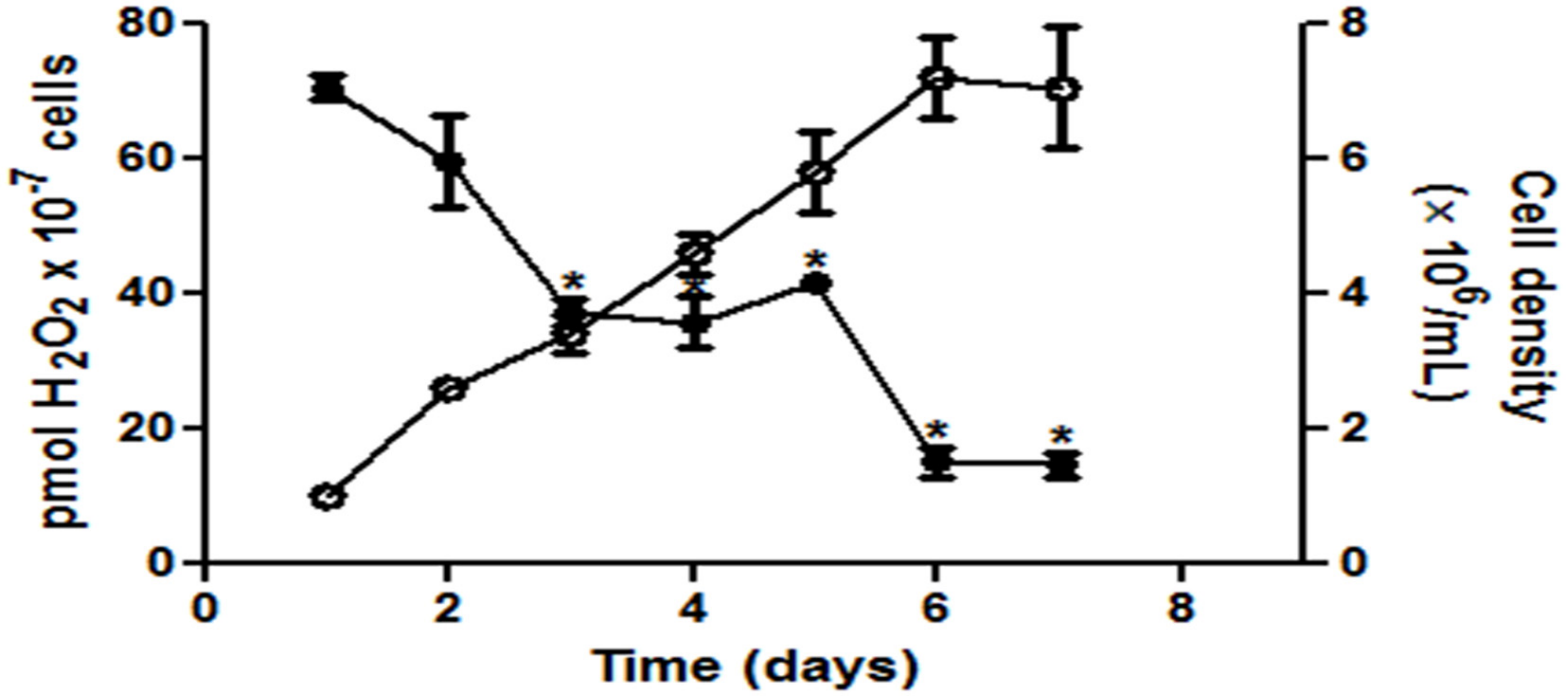
Hydrogen peroxide production during *L*. *amazonensis* proliferation days. The graph shows the hydrogen peroxide production (closed circles) and the number of cells present in the culture medium on each of the days (open circles). The values represent the mean ± standard error of at least three independent experiments. *Statistically significant when compared to day 1 (P < 0.05).

To verify if the presence of H_2_O_2_ could have a role in the proliferation of *Leishmania amazonensis* cells, we investigated the H_2_O_2_ generation profile during the growth curve of the parasite. There is an intense generation of H_2_O_2_ during the first days of the proliferation curve, decreasing over the days, and achieving minimum levels from the sixth day onward, when the cells reach their growth stationary phase (Fig 1).

### 2. Heme increases hydrogen peroxide production

**Fig 2:**
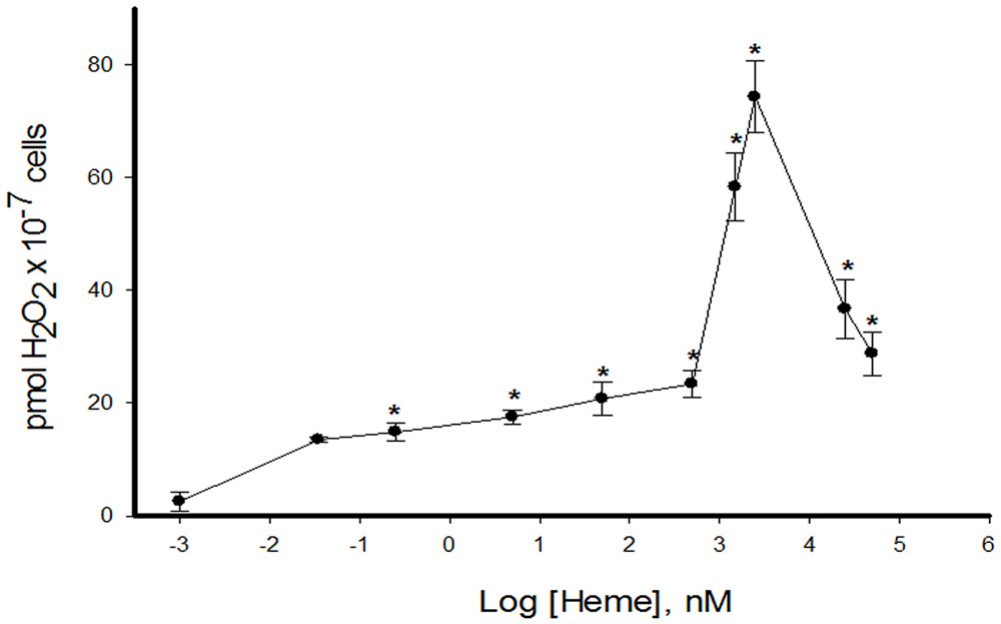
Effect of increasing concentrations of heme on the production of hydrogen peroxide by *L*. *amazonensis*. Living parasites were incubated for 20 min in the reaction medium with the addition of increasing concentrations of heme, as indicated on the abscissa. The values represent the mean ± standard error of at least three independent experiments. *Statistically significant when compared to control (P < 0.05).

To check whether heme, a molecule with pro-oxidant activity [38,39], was able to increase H_2_O_2_ production in *L*. *amazonensis* promastigotes, cells from sixth day of stationary phase, where the level of H_2_O_2_ is low, were incubated with increasing concentrations of heme. There is an increase in H_2_O_2_ levels in a dose response of heme concentration. The peak of H_2_O_2_ production (74 ± 6 pmol) occurs when cells were incubated in the presence of 2.5 μM heme. After this concentration, there is a pronounced drop in H_2_O_2_ generation (Fig 2).

### 3. H_2_O_2_ increases Na^+^/K^+^ ATPase activity

**Fig 3:**
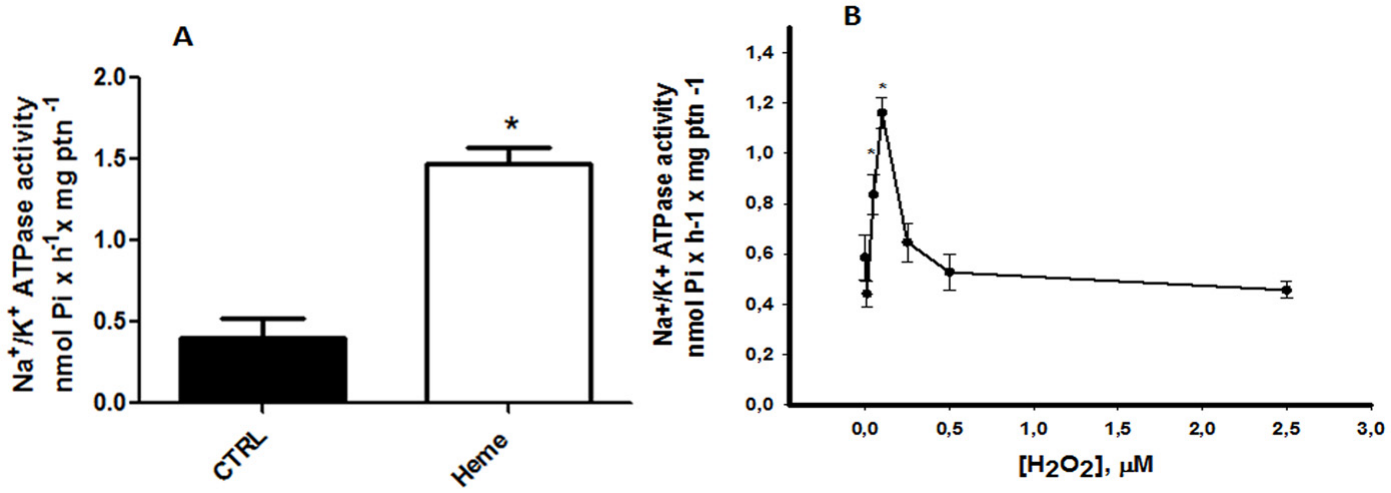
Effect of heme and hydrogen peroxide on the Na^+^/K^+^ ATPase activity in *L*. *amazonensis*. The intact cells were incubated with (white bar) or without (black bar) 2.5 μM heme (A) or with increasing concentrations of hydrogen peroxide (B) for 20 min. The values represent the mean ± standard error of at least three independent experiments. *Statistically significant when compared to control (P < 0.05). CTRL, control.

We have shown that heme activates Na^+^/K^+^ ATPase in *L*. *amazonensis* [25]. Incubating intact promastigotes with 2.5 μM heme, a concentration at which higher H_2_O_2_ levels were achieved, we confirmed the increase in Na^+^/K^+^ ATPase activity (Fig 3A). To investigate if H_2_O_2_ would be able to activate Na^+^/K^+^ ATPase, living promastigotes were incubated in the presence of increased concentrations of H_2_O_2_ and the Na^+^/K^+^ ATPase activity was assayed (Fig 3B). There is a sharp increase in Na^+^/K^+^ ATPase activity in response to low increased amounts of H_2_O_2_, with a peak of 1.16 ± 0.06 nmol Pi x h ^-1^ x mg protein^-1^ when the cells were incubated with 0.1 μM H_2_O_2_ (Fig 3B). Concentrations of H_2_O_2_ above this threshold caused a decrease in the activation of Na^+^/K^+^ ATPase activity, indicating a small window where H_2_O_2_ could work as a signaling molecule in the enzyme activity.

### 4. Treatment with 50 μM heme or 2.5 μM H_2_O_2_ did not affect *L*. *amazonensis* cellular viability

**Fig 4:**
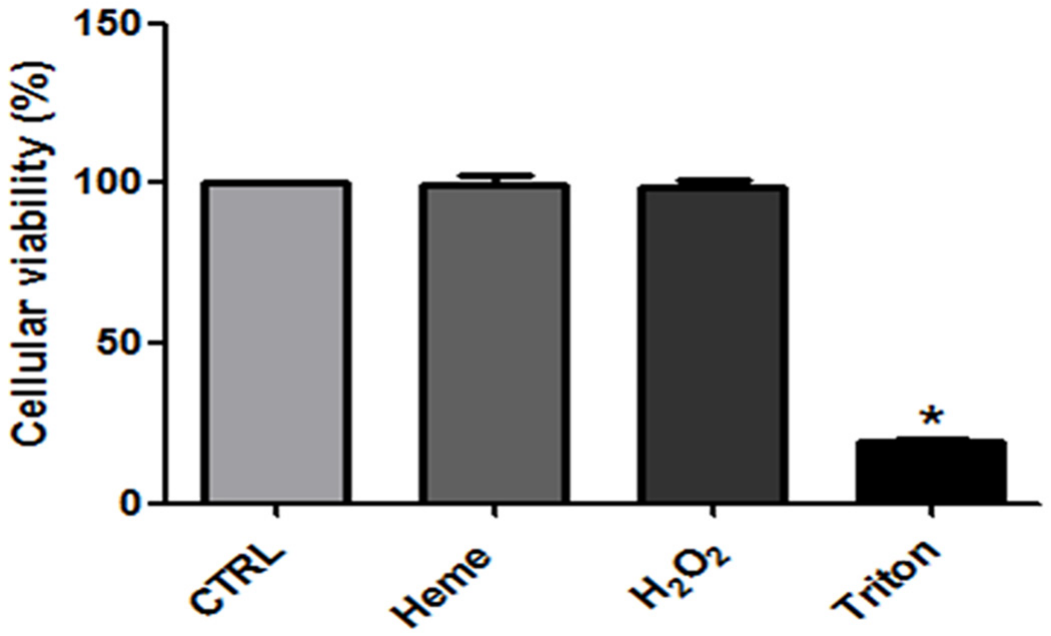
MTT assay for the viability of *L*. *amazonensis* incubated with 50 μM heme and 2.5 μM hydrogen peroxide. Living parasites were incubated for 1 hour in the reaction medium with the addition of 50 μM heme or 2.5 μM H_2_O_2_. Triton 1% was used as a positive control. The values represent the mean ± standard error of at least three independent experiments. *Statistically significant when compared to control (P <0.05). CTRL, control.

To investigate if incubation with heme or H_2_O_2_ affects cell viability, we used the highest concentration of the compounds tested and evaluated the cell integrity by the MTT assay. Neither 50 μM heme nor 2.5 μM H_2_O_2_ were able to affect cell viability when compared to control (Fig 4). Triton X-100 was used as a positive control for non-viable cells.

### 5. Comparison between H_2_O_2_ production and Na^+^/K^+^ ATPase activation promoted by heme in *L*. *amazonensis* from log and stationary phases

**Fig 5:**
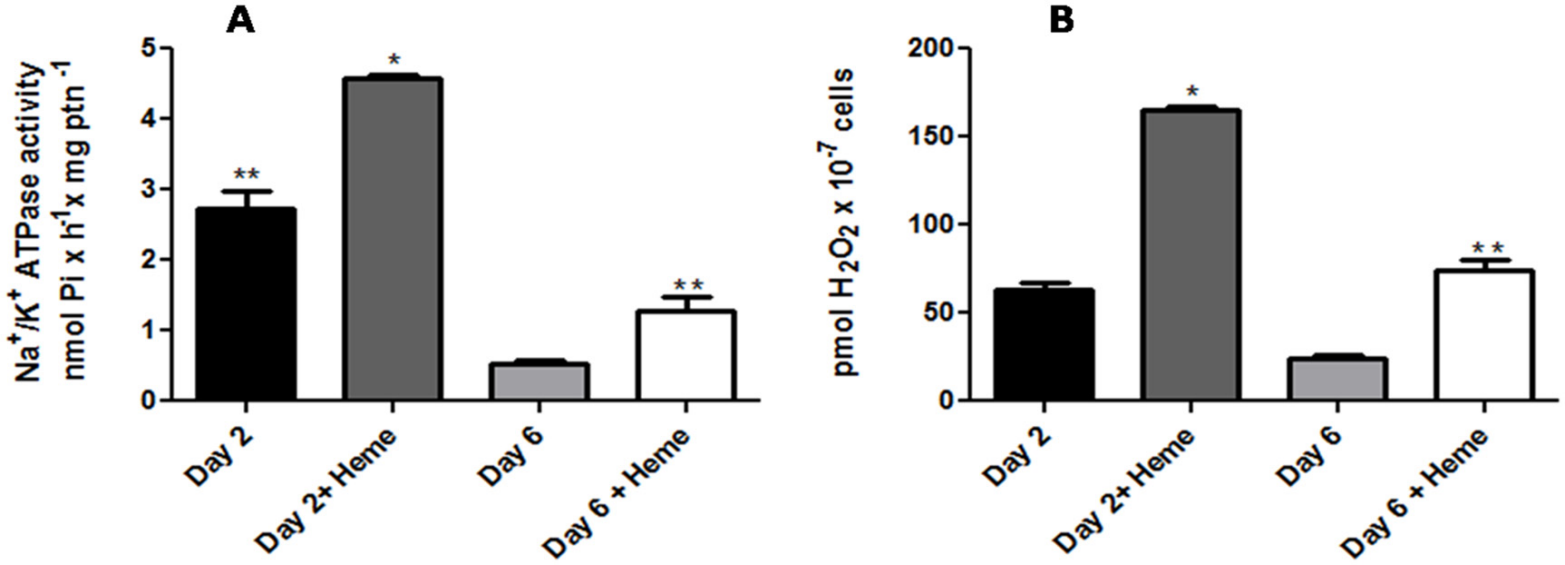
Hydrogen peroxide production and Na^+^/K^+^ ATPase activation promoted by heme in *L*. *amazonensis* at log and stationary phases. Living parasites on the second or sixth day of growth were incubated for 20 min in the reaction medium with or without the addition of 2.5 μM heme, as indicated on the abscissa. Na^+^/K^+^ ATPase activity (A) and hydrogen peroxide production (B) were determinated. The values represent the mean ± standard error of at least three independent experiments. *Statistically significant when compared to day 2. **Statistically significant when compared to day 6 (P < 0.05).

The generation of H_2_O_2_ is higher in the log phase than the stationary phase (Fig 1). Interestingly the Na^+^/K^+^ ATPase activity is also higher in the log phase than the stationary phase (Fig 5A). However, the heme stimulatory effects on Na^+^/K^+^ ATPase activity (Fig 5A) and H_2_O_2_ production (Fig 5B) in log and stationary phases were similar.

### 6. Effect of heme analogs, degradation products of heme, and FeCl_3_ on H_2_O_2_ production and Na^+^/K^+^ ATPase activity

**Fig 6:**
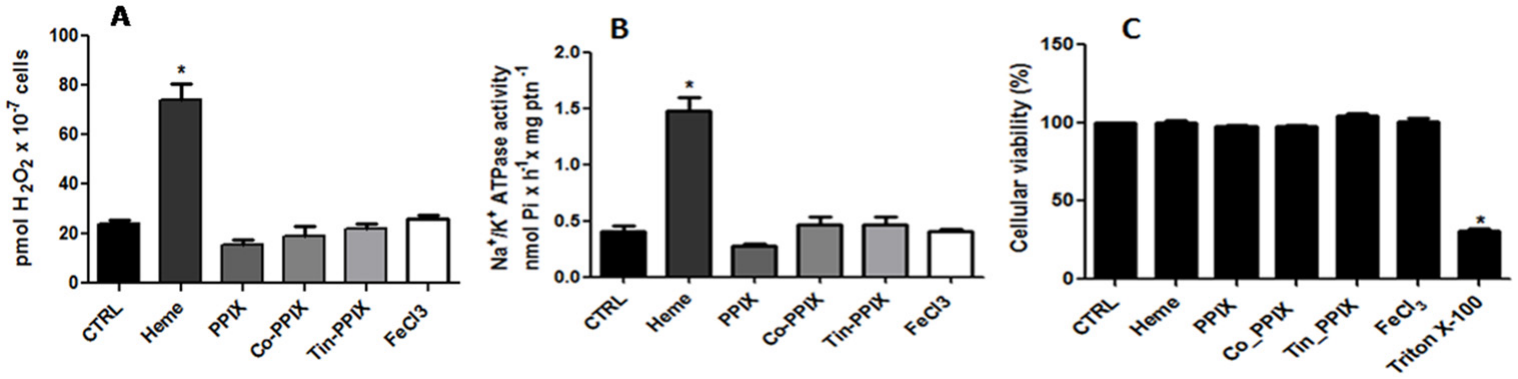
Effect of PPIX, Co-PPIX, Tin-PPIX and FeCl_3_ on the production of hydrogen peroxide by *L*. *amazonensis* and in the Na^+^/K^+^ ATPase activity of the parasite. Living parasites were incubated for 20 min in the reaction medium with the addition of 2.5 μM PPIX, 2.5 μM Co-PPIX, 2.5 μM Tin-PPIX or 2.5 μM FeCl_3_, as indicated on the abscissa. Hydrogen peroxide production (A), Na^+^/K^+^ ATPase activity (B) and Cellular viability (C) were determinated. The values represent the mean ± standard error of at least three independent experiments. *Statistically significant when compared to control (P < 0.05). CTRL, control.

**Fig 7:**
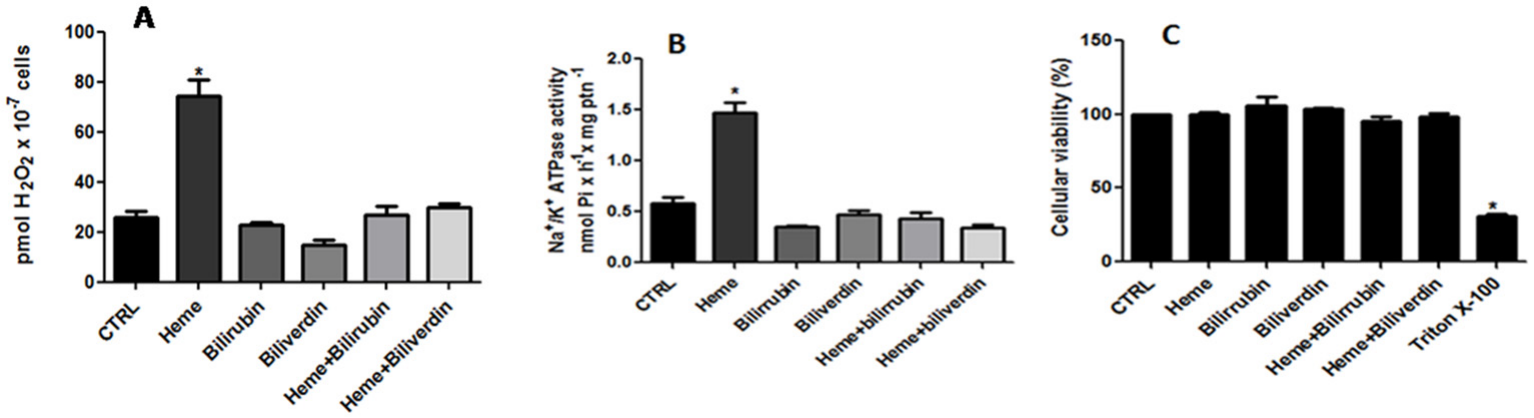
Effect of bilirubin and biliverdin on the production of hydrogen peroxide by *L*. *amazonensis* and in the Na^+^/K^+^ ATPase activity of the parasite. Living parasites were incubated for 20 min in the reaction medium with the addition of 2.5 μM bilirubin and 2.5 μM biliverdin or bilirubin and biliverdin plus 2.5 μM heme, as indicated on the abscissa. Hydrogen peroxide production (A), Na^+^/K^+^ ATPase activity (B) and Cellular viability (C) were determinated. The values represent the mean ± standard error of at least three independent experiments. *Statistically significant when compared to control (P < 0.05). CTRL, control.

To verify if the stimulatory effect on H_2_O_2_ production and Na^+^/K^+^ ATPase activity in *L*. *amazonensis* was caused exclusively by heme, we tested heme precursor, protoporphyrin IX (PPIX), two heme analogs, cobalt protoporphyrin IX (Co-PPIX) and tin-protoporphyrin IX (tin-PPIX), and FeCl_3_, (Fig 6A and 6B) at a concentration of 2.5 μM. We also tested the products of heme degradation, bilirubin and biliverdin (Fig 7A and 7B). None of the compounds that did not have effect on cell viability (Figs 6C and 7C), were able to mimic the stimulatory effect caused by heme on the production of H_2_O_2_ (Figs 6A and 7A) or the activation of Na^+^/K^+^ ATPase activity (Figs 6B and 7B). Moreover, addition of 2.5 μM bilirubin and biliverdin reverted the stimulatory effect of heme on H_2_O_2_ production and activation of Na^+^/K^+^ ATPase activity (Fig 7A and 7B).

### 7. Cell signaling involved in the production of H_2_O_2_ and on heme-dependent activation of Na^+^/K^+^ ATPase activity

**Fig 8:**
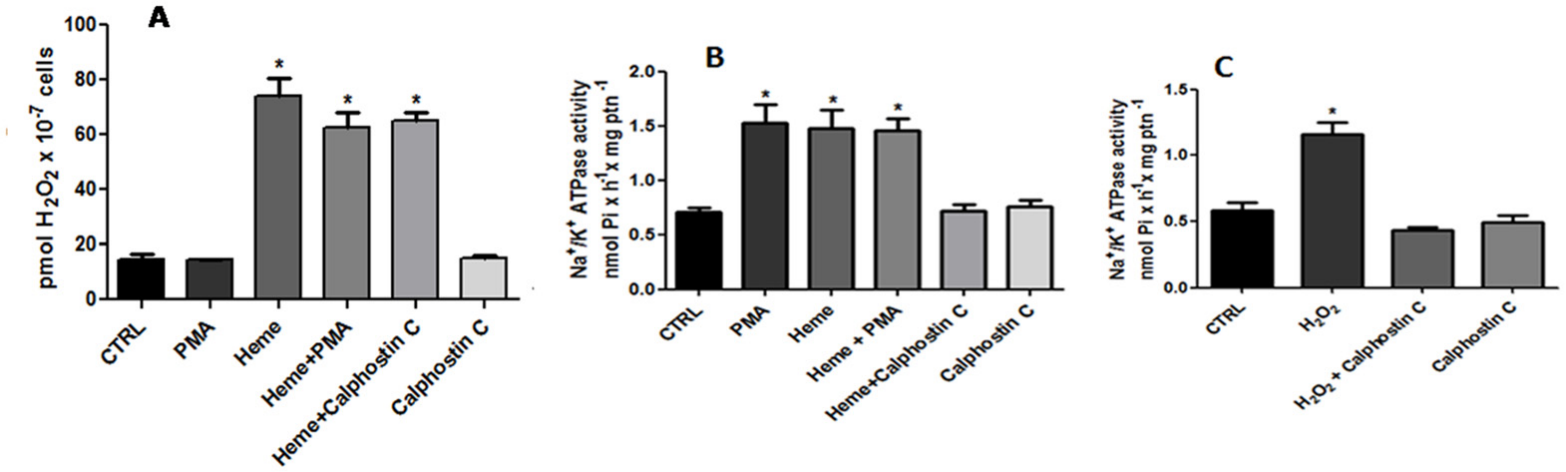
The involvement of PKC in the activation of Na^+^/K^+^ ATPase by hydrogen peroxide generated through theme. Living parasites were incubated for 20 min in the reaction medium with the addition of 0.1 nM PMA or 10 nM calphostin C in the presence or absence of 2.5 μM heme, as indicated on the abscissa. Hydrogen peroxide production (A) and Na^+^/K^+^ ATPase activity (B) were determined. Intact cells were incubated with 10 nM calphostin C for 20 min, either with or without the addition of 0.1 μM hydrogen peroxide, as indicated on the abscissa, for an additional 20 min. Na^+^/K^+^ ATPase activity (C) was determined. The values represent the mean ± standard error of at least three independent experiments. *Statistically significant when compared to control (P < 0.05). CTRL, control.

PKC was shown to be involved in the activation of Na^+^/K^+^ ATPase by heme [25]. In this sense, we investigated if phorbol ester (PMA), a potent tumor promoter that mimics diacylglycerol (DAG) in activation of PKC [40], and calphostin C, a specific and potent inhibitor of PKC [41,42], were able to modulate heme-dependent activation of Na^+^/K^+^ ATPase activity and the increase of H_2_O_2_ production. No effect of PMA and calphostin C could be observed on H_2_O_2_ generation (Fig 8A). However, PMA was able to activate the Na^+^/K^+^ ATPase activity (Fig 8B). In addition, calphostin C suppressed the activation of Na^+^/K^+^ ATPase activity promoted by Heme (Fig 8B) and also abolished the activation of Na^+^/ K^+^ ATPase activity promoted by H_2_O_2_ (Fig 8C). These data could be suggesting that H_2_O_2_-dependent activation of Na^+^/ K^+^ ATPase activity would be through PKC activity.

### 8. H_2_O_2_ stimulates PKC activity

**Fig 9:**
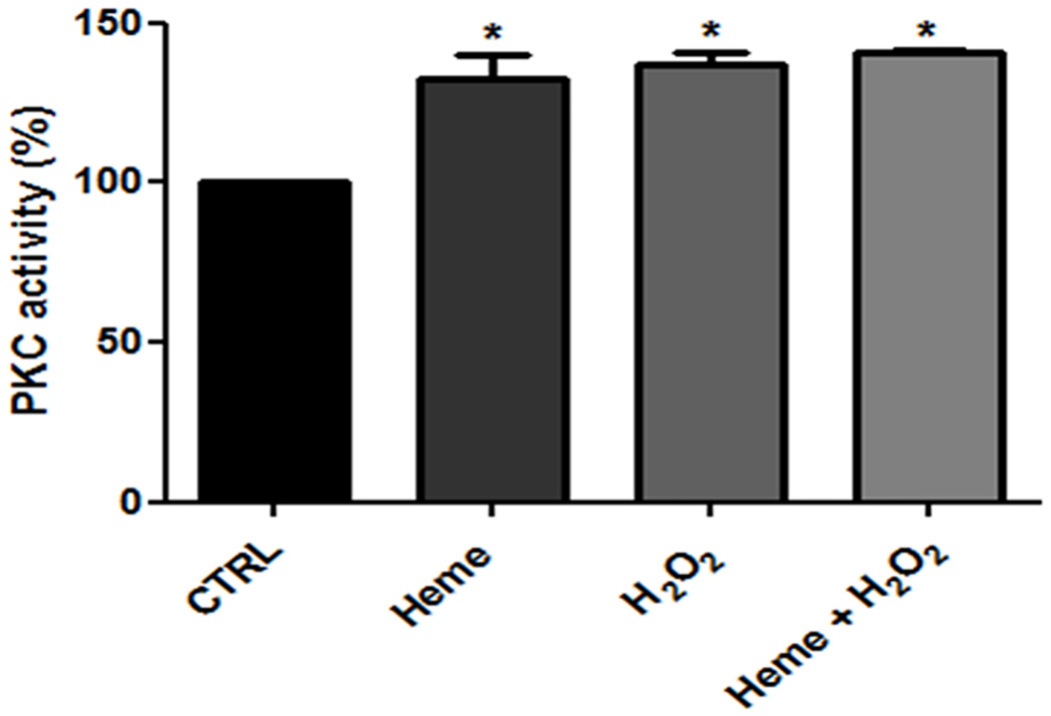
Effect of hydrogen peroxide on the PKC activity in *L*. *amazonensis*. Intact promastigotes were incubated for 20 minutes in a reaction medium containing 2.5 M heme or 0.1 μM hydrogen peroxide, or both compounds together, as indicated on the abscissa. The data indicate mean enzyme activity ± SE of at least three experiments, each with different cell suspensions. *Statistically significant when compared to control (n = 3, P < 0.05). CTRL, control.

To confirm our hypothesis that in *L*. *amazonensis* PKC is activated by H_2_O_2_, we evaluated the PKC activity in the presence of heme, H_2_O_2_ and heme plus H_2_O_2_. As expected, we observed a similar increase in PKC activity in the presence of both compounds, and there was no additive effects when cells were incubated with heme plus H_2_O_2_ (Fig 9).

### 9. The effect of PEG-catalase on the Na^+^/K^+^ ATPase activity

**Fig 10:**
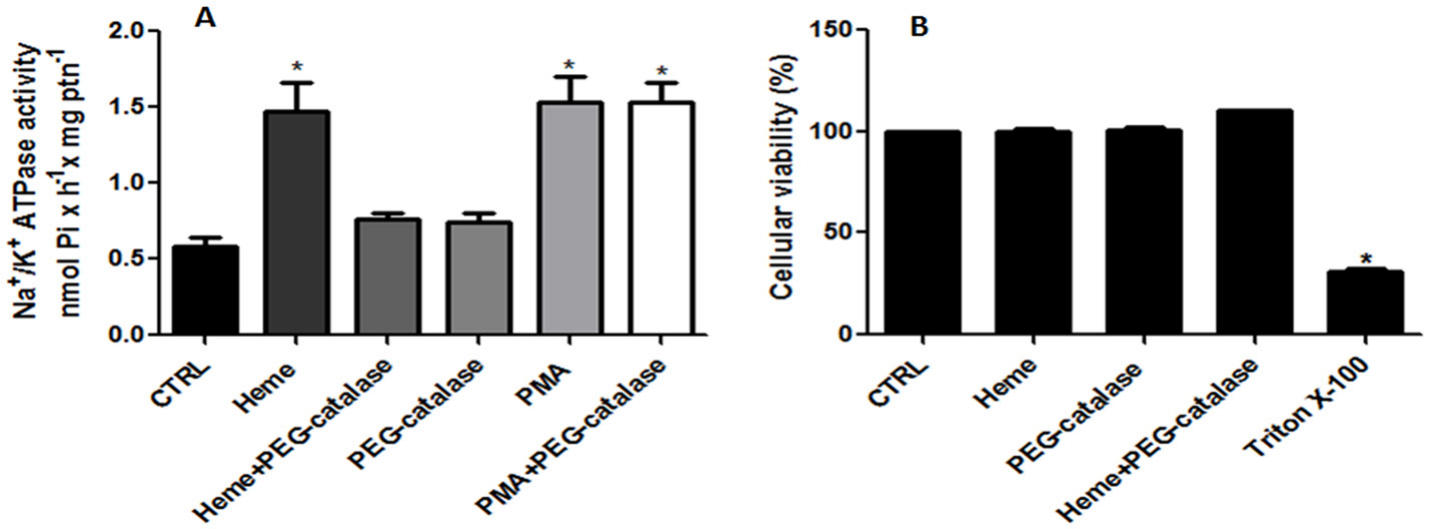
Effect of PEG-catalase on the Na^+^/K^+^ ATPase activity in *L*. *amazonensis*. The intact cells were incubated with 300U/mL PEG-catalase for 20 min and then either treated or not treated with 2.5 μM heme or 0.1 nM PMA, as indicated on the abscissa, for an additional 20 min. Na^+^/K^+^ ATPase activity (A) and Cellular viability (B) were determinated. The values represent the mean ± standard error of at least three independent experiments.*Statistically significant when compared to control (n = 3, P < 0.05). CTRL, control.

To confirm that Na^+^/K^+^ ATPase stimulation by heme is through H_2_O_2_, we used catalase-polyethylene glycol (PEG-catalase), which is a scavenger of H_2_O_2_ that can cross the plasma membrane. The stimulatory effect of heme on Na^+^/K^+^ ATPase activity was abolished in the presence of the PEG-catalase; however, as expected, the same was not seen when PMA was used (Fig 10A). While PMA activates PKC directly, heme seems to activate PKC through H_2_O_2_ generation. The treatment with PEG-catalase did not have effect on cell viability (Fig 10B)

### 10. The effect of Mito-TEMPO on the production of H_2_O_2_ and the heme-dependent activation of Na^+^/K^+^ ATPase activity

**Fig 11:**
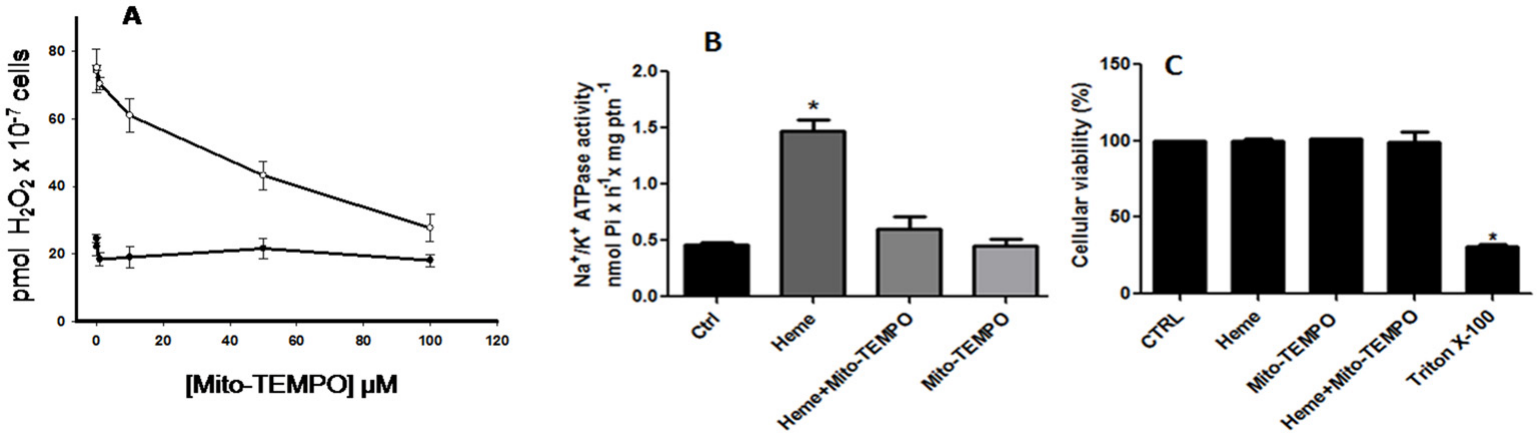
Effect of Mito-TEMPO on the production of hydrogen peroxide by *L*. *amazonensis* and on the Na^+^/K^+^ ATPase activity of the parasite. Living parasites were incubated for 20 min in the reaction medium, with the addition of increasing concentrations of Mito-TEMPO in the presence (white circles) or absence (black circles) of 2.5 μM heme, as indicated on the abscissa (A). The intact cells were incubated with 100 μM Mito-TEMPO, in the presence or absence of 2.5 μM of heme, as indicated on the abscissa, for 20 min. Na^+^/K^+^ ATPase activity (B) and Cellular viability (C) were determinated. The values represent the mean ± standard error of at least three independent experiments. *Statistically significant when compared to cells incubated with 2.5 μM of heme without Mito-TEMPO (n = 3, P < 0.05).

To investigate where heme-dependent H_2_O_2_ production was coming from, we used Mito-TEMPO, a mitochondria-targeted SOD mimetic that also reduces mitochondria electron leak and inhibits the production of all ROS, including H_2_O_2_ [43,44]. Promastigotes cells were incubated with increasing concentrations of Mito-TEMPO and the production of H_2_O_2_ was evaluated (Fig 11A). At 100 μM of Mito-TEMPO the production of H_2_O_2_ stimulated by heme was the same as control (without heme). Mito-TEMPO was also capable of abolishing the stimulatory effect of heme on the Na^+^/K^+^ ATPase activity (Fig 11B). In this condition, Mito-TEMPO did not interfered on cell viability (Fig 11C).

## Discussion

Recent studies have demonstrated that H_2_O_2_ could play a key role in intracellular signal transduction through the reversible activation / inactivation of the active site of several enzymes [12, 14, 45, 46]. Protozoan parasites of genus Tryoanosoma are able to generate H_2_O_2_ during its growth [12]. High levels of H_2_O_2_ produced at log phase of growth of *Leishmania amazonensis* (Fig 1) could be indicating that this molecule is important to parasite to grow.

Knowledge of signal transduction pathways in protozoan parasites is extremely important, considering that understanding the cellular physiology of these organisms can help the design of new drugs and the understanding of the interaction with its host. Felibertt *et al* [26] proposed in 1995 that the Na^+^/K^+^ ATPase activity could be involved in the generation of an electrochemical gradient of Na^+^ and K^+^ in *L*. *mexicana*. The Na^+^ gradient gives the energy required for the transport of nutrients and other solutes. A signaling pathway activated by heme involving phosphatidylinositol-specific phospholipase C (PI-PLC) and PKC, which leads the activation of Na^+^/ K^+^ ATPase, has been described in *L*. *amazonensis* [25].

*Leishmania* PKC-like has been associated with biological properties such as interaction with host macrophages [30,47] and maintenance of ion homeostasis [25, 27, 30]. Free heme, an amphipathic porphyrin containing iron, can catalyze the production of reactive oxygen species [6–9]. In this work, we show that promastigotes of *L*. *amazonensis* in the presence of 2.5 μM heme have a spike in the production of H_2_O_2_, reaching an amount of 74 ± 6 pmol (Fig 2). It is currently accepted that organisms have not only adapted to deal with oxidizing species but also developed mechanisms to make use of these free radicals [10]. It has been reported that ROS generated by heme activates the intestinal epithelial cells through activation of focal adhesion kinase (FAK) and the polymerization of the actin cytoskeleton [9]. In *T*. *brucei* and *T*. *cruzi*, respectively, heme-dependent ROS production was shown to modulate ecto-nucleoside triphosphate diphosphohydrolase (E-NTPDase) activity [8] and Ca^2+^ calmodulin kinase II activity (CaMKII-like), which activates epimastigote proliferation [48]. Thus, ROS may act as second messengers, which are required in various signaling cascades [11]. In this context, we aimed to investigate if H_2_O_2_ could have a role in the heme-dependent stimulation of Na^+^/K^+^ ATPase activity (Fig 3A). The maximum activity, 1.16 ± 0.06 nmol Pi x h^-1^ x mg^-1^, was at a concentration of 0.1 μM H_2_O_2_, nearly a 2-fold increase from control (Fig 3B). None of the concentrations of heme and H_2_O_2_ tested affected cell viability (Fig 4).

We also evaluated the heme stimulatory effect on the Na^+^/K^+^ ATPase activity and on the H_2_O_2_ production at log and stationary phases of *L*. *amazonensis* and no difference was observed (Fig 5A and 5B). Interestingly the Na^+^/K^+^ ATPase activity is highest in log phase of growth, probably because high levels of H_2_O_2_ are found (Fig 5A and 5B).

Recently, a correlation between iron uptake by ferrous iron transporter (LIT1) in *L*. *amazonensis* and an intracellular H_2_O_2_ increase was described [49]. In aerobic environments ferrous iron (Fe^2+^) is easily oxidized to the ferric form (Fe^3+^), but to cross the plasma membrane Fe^3+^ must be converted to Fe^2+^, the LIT1 substrate, by a ferric iron reductase [50]. In this context, and knowing that heme is a source of iron, we tested ferric chloride for the ability to induce the production of H_2_O_2_ and activate the Na^+^/K^+^ ATPase (Fig 6A and 6B). Ferric chloride was not able to mimic the action of heme in the production of H_2_O_2_ or in Na^+^/K^+^ ATPase activity. To evaluate if the signaling events occur exclusively in the presence of heme, we also tested protoporphyrin IX, cobalt protoporphyrin and tin-protoporphyrin (Fig 6A and 6B). The stimulatory effect of heme in the production of H_2_O_2_ or in Na^+^/K^+^ ATPase activity were not observed in the presence of these compounds.

It has been described in other models that biliverdin and bilirubin possess antioxidant activity [51,52]. In this work promastigote cells were incubated with heme in the presence or absence of bilirubin or biliverdin and tested for the ability to induce the production of H_2_O_2_ (Fig 7A) and activate the Na^+^/K^+^ ATPase (Fig 7B). The effect of heme was abolished in the presence of bilirubin and biliverdin, showing that in *L*. *amazonensis* they also have antioxidant activity.

In *L*. *amazonensis* PKC is involved in the pathway that leads to activation of Na^+^/K^+^ ATPase by heme [25]; however, until this work, there was no evidence of PKC modulation by hydrogen peroxide in trypanosomatids. In neutrophils and macrophages it was shown that PKC was involved in increasing ROS levels [53,54], but in this work we show that modulators of PKC activity, such as the activator PMA or the inhibitor calphostin C, were not able to modify the increased levels of H_2_O_2_ triggered by heme, excluding PKC participation in this process (Fig 8A). On the other hand, Na^+^/K^+^ ATPase activity increased in the presence of PMA, and the increase in activity induced by heme is abolished in the presence of calphostin C (Fig 8B). We also tested the ability of H_2_O_2_ to stimulate Na^+^/K^+^ ATPase activity when PKC is inhibited with calphostin C (Fig 8C), confirming that H_2_O_2_ does not activate Na^+^/K^+^ ATPase directly, but through PKC activation (Fig 9). Activation of PKC by ROS has been shown in several other models. In hepatocytes mitochondrial ROS generation after cell exposure to sodium arsenite (NaAsO2) was shown to induce PKC activation, which in turn activates c-Jun *N*-terminal kinases (JNK), leading to the progression of apoptosis [55]. In splenic lymphocytes, radiotherapy treatment led to a decrease in the activities of antioxidant enzymes and an increase in cell oxidative damage. This effect was followed by an increase in PKC activity and activation of signaling with a cytoprotective effect [56].

To ensure that hydrogen peroxide generated by incubation with heme would be involved in the modulation of Na^+^/K^+^ ATPase activity, we used PEG-catalase (Fig 10) and mito-TEMPO (Fig 11), two known antioxidants. Both compounds were able to abolish the heme effect, showing that this process is dependent on an oxidation effect and that mitochondria are the most likely source of increased H_2_O_2_.

**Fig 12:**
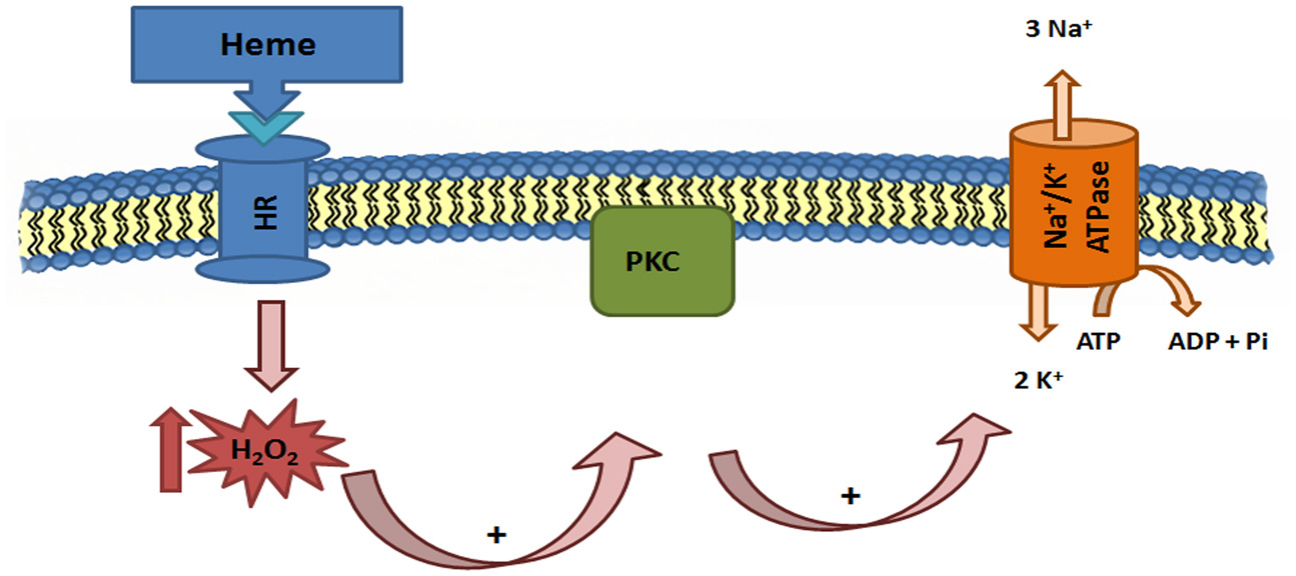
Hypothetical signaling pathway of Na^+^/K^+^ ATPase activation by increasing H_2_O_2_ production through heme and stimulation of the PKC in *Leishmania amazonensis* promastigotes. HR, heme receptor; H2O2, hydrogen peroxide; PKC, protein kinase C; ATP, adenosine triphosphate; ADP, adenosine diphosphate; Pi, inorganic phosphate.

Taken together, our results show a signaling pathway that is activated by heme, causing an increase in the intracellular H_2_O_2_, which leads to the activation of PKC, culminating in the increase of Na^+^/K^+^ ATPase activity (Fig 12). Further investigation would be necessary to explain if heme signalization occurs through a receptor located on the promastigote plasma membrane or if heme is transported into the cell.
